# Performance comparison of ventricular and arterial dP/dt_max_ for assessing left ventricular systolic function during different experimental loading and contractile conditions

**DOI:** 10.1186/s13054-018-2260-1

**Published:** 2018-11-29

**Authors:** Manuel Ignacio Monge Garcia, Zhongping Jian, Jos J. Settels, Charles Hunley, Maurizio Cecconi, Feras Hatib, Michael R. Pinsky

**Affiliations:** 1 Unidad de Cuidados Intensivos, Hospital Universitario SAS de Jerez, C/ Circunvalación, s/n, 11407 Jerez de la Frontera, Spain; 2 Edwards Lifesciences, Irvine, California USA; 3 Orlando Regional Medical Center, Orlando Health, Florida, USA; 4 Department Anaesthesia and Intensive Care Units, Humanitas Research Hospital, Humanitas University, Milan, Italy; 5 Department of Critical Care Medicine, University of Pittsburgh School of Medicine, Pittsburgh, USA

**Keywords:** dP/dt_max_, Contractility, Arterial pressure, Left ventricular function

## Abstract

**Background:**

Maximal left ventricular (LV) pressure rise (LV dP/dt_max_), a classical marker of LV systolic function, requires LV catheterization, thus surrogate arterial pressure waveform measures have been proposed. We compared LV and arterial (femoral and radial) dP/dt_max_ to the slope of the LV end-systolic pressure-volume relationship (Ees), a load-independent measure of LV contractility, to determine the interactions between dP/dt_max_ and Ees as loading and LV contractility varied.

**Methods:**

We measured LV pressure-volume data using a conductance catheter and femoral and radial arterial pressures using a fluid-filled catheter in 10 anesthetized pigs. Ees was calculated as the slope of the end-systolic pressure-volume relationship during a transient inferior vena cava occlusion. Afterload was assessed by the effective arterial elastance. The experimental protocol consisted of sequentially changing afterload (phenylephrine/nitroprusside), preload (bleeding/fluid bolus), and contractility (esmolol/dobutamine). A linear-mixed analysis was used to assess the contribution of cardiac (Ees, end-diastolic volume, effective arterial elastance, heart rate, preload-dependency) and arterial factors (total vascular resistance and arterial compliance) to LV and arterial dP/dt_max_.

**Results:**

Both LV and arterial dP/dt_max_ allowed the tracking of Ees changes, especially during afterload and contractility changes, although arterial dP/dt_max_ was lower compared to LV dP/dt_max_ (bias 732 ± 539 mmHg⋅s^− 1^ for femoral dP/dt_max_, and 625 ± 501 mmHg⋅s^− 1^ for radial dP/dt_max_). Changes in cardiac contractility (Ees) were the main determinant of LV and arterial dP/dt_max_ changes.

**Conclusion:**

Although arterial dP/dt_max_ is a complex function of central and peripheral arterial factors, radial and particularly femoral dP/dt_max_ allowed reasonably good tracking of LV contractility changes as loading and inotropic conditions varied.

**Electronic supplementary material:**

The online version of this article (10.1186/s13054-018-2260-1) contains supplementary material, which is available to authorized users.

## Background

Left ventricular (LV) contractility is one of the main determinants of cardiac function and an important element of the hemodynamic evaluation of the critically ill [1]. Impaired LV contractility is frequently seen in patients with acute coronary syndrome and sepsis [2]. Although LV end-systolic elastance (Ees) is the reference method for assessing LV contractility [3], its bedside use is limited by its invasiveness and the technical difficulties associated with its estimation [4]. LV ejection fraction (EF_LV_), estimated by echocardiography as the fractional area of contraction, is currently the most used clinical index for estimating LV systolic function. However, EF_LV_ has known limitations as an index of cardiac inotropy, such as the high dependency on the cardiac loading conditions [5, 6]. Although new echocardiographic indexes, such as speckle-tracking-derived LV global longitudinal strain or strain rate, have been recently introduced [7], their need of sophisticated software and trained operators precludes their use for continuous hemodynamic monitoring of the LV systolic function.

The maximum rate of LV pressure during isovolumetric contraction (LV dP/dt_max_) has been classically considered as a marker of LV inotropic state [8]. However, as LV dP/dt_max_ requires a direct measure of LV pressure, other surrogates have been proposed using the arterial pressure waveform. Peripheral dP/dt_max_, as measured from catheters inserted into the femoral or radial arteries have been suggested as feasible surrogates for LV dP/dt_max_ [9, 10]. However, as the arterial pressure results from the combined interaction of the LV ejection and the arterial system properties, other potential factors could also contribute to the peripheral dP/dt_max_, degrading its accuracy as a measure of LV contractile state [10, 11].

To address this issue, we compared LV and peripheral dP/dt_max_ during different preloading and afterloading and contractility conditions against the LV end-systolic elastance, a load-independent measure of cardiac contractility, and the other cardiac and arterial factors that were influencing these parameters in our established porcine model.

## Methods

The study was approved by the Institutional Animal Care and Use Committee (IACUC) at the Edwards Research Center, and all experimentation was performed in accordance with the USDA Animal Welfare Act regulations (AWArs), and the Guide for the Care and Use of Laboratory Animals (ILAR, NAP, Washington, DC, 2010, 8th edition). The Test Facility is accredited by the Association for the Assessment and Accreditation of Laboratory Animal Care, International (AAALACi) and registered with the United States Department of Agriculture to conduct research with laboratory animals. The Animal Research: Reporting of In Vivo Experiments (ARRIVE) guidelines were used for the elaboration of this manuscript [12].

Ten females adult Yorkshire cross breed pigs weighing 81 ± 6 kg were studied. They were maintained in temperature-controlled and humidity-controlled rooms with a typical light–dark cycle and given standard chow and tap water ad libitum. Prior to anesthesia induction, a general physical examination was performed including weight, temperature, heart rate, respiratory rate, mucus membrane, capillary refill time, general condition, and heart and lung auscultation. If found to be stable, the animal then was premedicated with an intramuscular combination of telazol (4.4 mg⋅kg^− 1^), ketamine (2.2 mg⋅kg^− 1^) and xylazine (1.1 mg⋅kg^− 1^). Once the animal was on the preparation table, an endotracheal tube was placed in the trachea, and was anesthetized by gas with a mixture of 3–4% isoflurane and 100% oxygen. An intravenous catheter was placed in the auricular artery and vein and the neck and inguinal areas were shaved and cleaned, and the electrocardiogram (EKG) electrodes applied. Once on the operating table the pig was mechanically ventilated in a volume-controlled mode with respiratory rate set at 13–15 cycles⋅min^− 1^, tidal volume at 10 ml⋅kg^− 1^ (plus 100 ml compensation for dead space), and anesthesia was maintained with isoflurane 1.5–2.5% and a mixture of oxygen, air and/or nitrous oxide and fraction of inspired oxygen (FiO_2_) of 60–80%. Fluid was maintained by an intravenous infusion of Ringer’s lactate solution (2–4 ml⋅kg^− 1^⋅h^− 1^). Rectal temperature was monitored and kept between 36 and 37 °C using a heating pad. Animal anesthesia were monitored and recorded approximately every 15 min for the duration of the experimentation. Anesthesia depth and pain were assessed throughout the study by performing jaw tone and toe pinch. Positive jaw tone and negative toe pinch meant that the animal was under a non-painful depth of anesthesia. No paralytic agents were used for this study.

Instantaneous LV pressure-volume (PV) measurements were obtained from a 7Fr-lumen dual-field catheter with 12-equidistant electrodes and a high-fidelity pressure sensor (CA71083PL, CD Leycom, Zoetermeer, the Netherlands) connected to a PV signal processor (Inca®, CD Leycom, Zoetermeer, the Netherlands). The catheter tip was positioned in the LV apex and the correct placement was confirmed by fluoroscopy and the examination of the segmental LV PV loops.

### Data collection and analysis

Volume signal calibration was performed via right-side heart catheterization with a Swan-Ganz catheter in the pulmonary artery (Vigilance, Edwards Lifesciences, Irvine, CA, USA). Volume signal calibration comprised 3–5 thermodilution boluses for the determination of cardiac output (CO). Correction for parallel conductance (the conductance of the surrounding tissues, which was subtracted from the raw catheter volume) was performed with the injection of 10-ml boluses of 5% hypertonic saline through the distal port of the pulmonary artery catheter. The conductance signals obtained were then converted to calibrated volume signals by considering the inter-electrode spacing, the parallel conductance correction and the CO calibration factor obtained from thermodilution [13, 14]. CO calibration and parallel conductance correction were performed before starting the experimental protocol and after the fluid bolus stage.

**Fig. 1:**
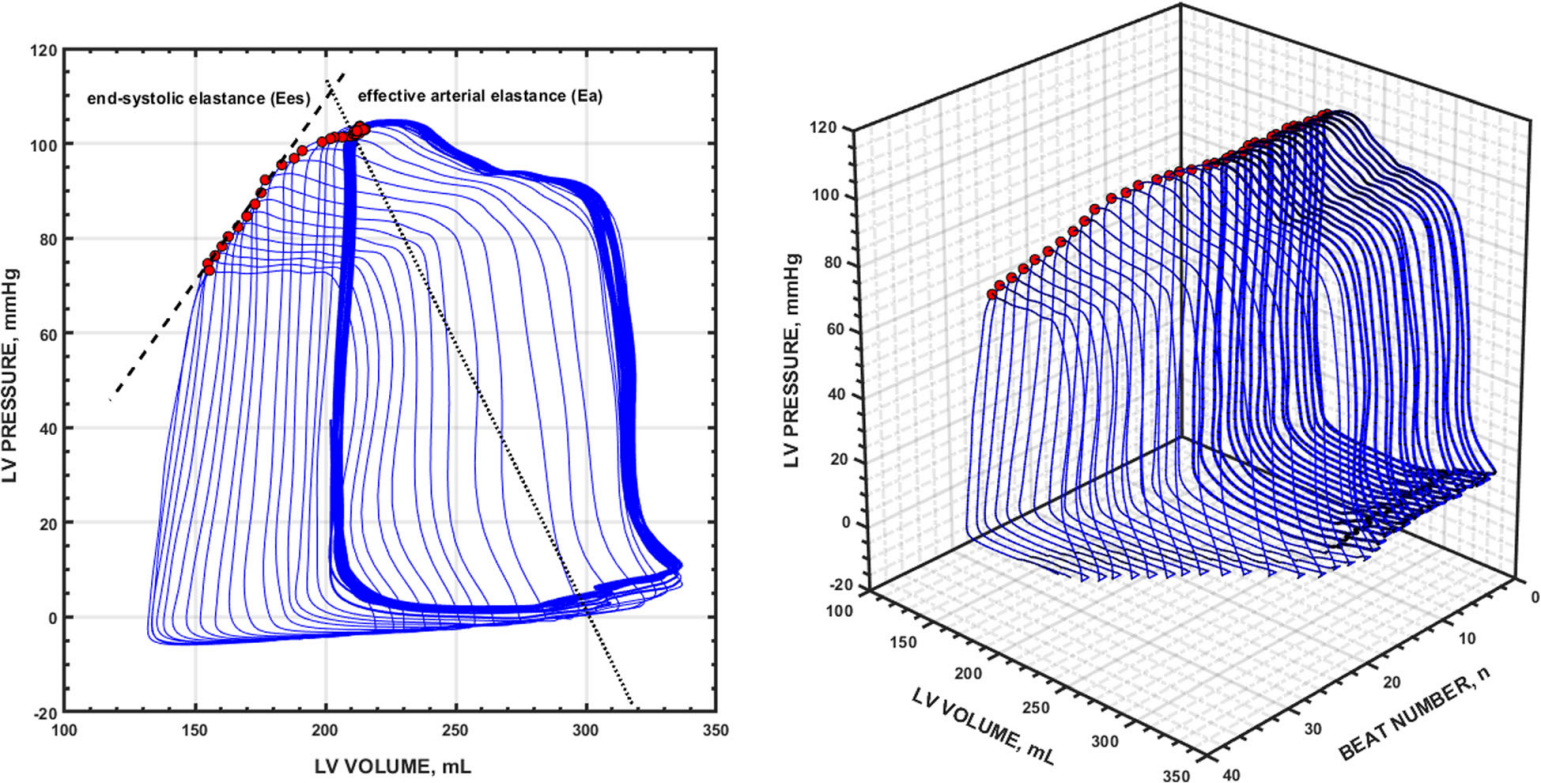
Example of the left ventricular pressure-volume (PV) analysis during an inferior vena cava occlusion. Two-dimensional (left) and 3d representation (right) of the left ventricular PV loops during a transient inferior vena cava (IVC) occlusion. Red points represent the maximal elastance for each cardiac cycle. The slope of these maximum elastance values obtained during a transient decrease in preload represents the left ventricle (LV) end-systolic elastance (Ees, a marker of LV contractility). The outlined PV loops represent the cardiac cycles obtained just before the IVC during an end-expiratory pause for measuring end-systolic pressure and effective arterial elastance (Ea, a net measure of LV afterload). Dashed line connects the maximal elastance during the IVC maneuver and represents the end-systolic elastance (Ees). The dotted line connects the end-systolic pressure with the stroke volume (defined by the width of the PV loop: end-diastolic volume minus end-systolic volume) representing the effective arterial elastance (Ea)

LV pressure-volume data acquisition and analysis were performed in a dedicated software system (Conduct NT, version 3.18.1, CD Leycom, Zoetermeer, the Netherlands). The signals were recorded at 250 Hz sampling rate and filtered using a 25 Hz low-pass filter. Before and after each experimental stage, three transient (15 s maximum) occlusions of the inferior vena cava (IVC) were performed during apnea using a Fogarty balloon. This procedure was repeated if ectopic beats were detected. End-systolic pressure (Pes), stroke volume (SV), CO, end-diastolic and end-systolic volumes (EDV and ESV, respectively), end-diastolic pressure, left ventricular ejection fraction (LVEF), effective arterial elastance (Ea) (a lumped parameter of LV afterload calculated as Ea = Pes/SV [15]), arterial (radial and femoral) and LV dP/dt_max_ were calculated from 3 to 5 beats in steady-state conditions during the respiratory pause just before the IVC occlusion. Ees was determined as the slope of the end-systolic pressure-volume relationship during the first 10 s of the IVC occlusion, calculated from the linear regression analysis of the maximal elastance (E) points on each cardiac cycle, defined as E(t) = P(t)/V(t) – V_0_, where V_0_ is the volume-axis intercept or the LV unstressed volume [13]. An example of a typical PV loop analysis is illustrated in Fig. 1.

The radial and femoral arterial pressure waveform were continuously recorded with a fluid-filled pressure transducer (FloTracIQ sensor; Edwards Lifesciences, Irvine, CA, USA) at a sampling rate of 100 Hz, using an EV1000 monitor (Edwards Lifesciences, Irvine, CA, USA) and then transferred onto a computer. Throughout the study, optimal damping of the arterial pressure waveform was carefully checked by fast flushing the line and checking the square wave test. Then cardiac beats were detected and maximum dP/dt for each beat was then calculated. The maximum dP/dt for all beats in a 20-s window of waveform was then averaged and the mean of three consecutive values (corresponding to measurement in 1 min) was used for analysis, in order to minimize the impact of respiratory variations on arterial pressure. The arterial system was characterized by a two-element Windkessel model [16], that consists of a resistive component: the total vascular resistance (TVR) = femoral mean arterial pressure (MAP)/CO * 80; and a pulsatile component: lumped arterial compliance (C_art_) = SV/local arterial pulse pressure (femoral or radial pulse pressure) [17]. Preload-dependency was continuously estimated using femoral pulse contour-derived stroke volume variation (SVV).

### Experimental protocol

Before starting with the experimental protocol, the animal received a fluid bolus (Voluven®, 130/0.4, Fresenius Kabi Deutschland GmbH, Bad Homburg, Germany) until it reached a SVV value < 15% for preload-optimization. Then they were allowed to stabilize for at least 10 min (heart rate and MAP variation < 5%). The study protocol consisted of three consecutive stages with two opposite (up and down) interventions each: changes in afterload (phenylephrine and nitroprusside), preload (bleeding and fluid bolus), and contractility (esmolol and dobutamine). The experiment started with the afterload interventions: the pigs were treated with sodium nitroprusside at a concentration of 100–200 mg⋅kg^− 1^⋅min^− 1^ to decrease MAP to 40% from baseline (but not below 50 mmHg, allowing for adequate hemodynamic tolerance during the IVC occlusions) followed by recovery to baseline status. Then they were treated with a phenylephrine infusion to increase MAP by 40% mmHg from baseline (30–120 mg⋅kg^− 1^⋅min^− 1^) and were allowed to recover. Subsequently, for preload interventions, the animals were submitted to stepwise bleeding of 12 ml⋅kg^− 1^ at the rate of 50 ml⋅min^− 1^ and the blood was stored in a heparinized sterile bag. Then the blood was slowly reinfused (50 ml⋅min^− 1^), and a fluid bolus of 10 ml⋅kg^− 1^ of colloid in 5 min was infused. After the fluid administration, the contractility interventions followed: an esmolol infusion was introduced at 50 μg⋅kg^− 1^⋅min^− 1^ and was increased until LV dP/dt_max_ was decreased by 50% from its previous value, with a limit dose of 200 μg⋅Kg^− 1^⋅min^− 1^. Then the esmolol infusion was stopped and, after a period of recovery, the animals were treated with a dobutamine infusion (5 μg⋅kg^− 1^⋅min^− 1^) to increase LV dP/dt_max_ by 50%. LV PV loops and arterial pressure waveforms were obtained during baselines and after each intervention stage.

### Statistical analysis

Data are expressed as the mean (SD) or median (25th to 75th interquartile range), as appropriate. The normality of data was checked by the Shapiro-Wilk test. Since we were interested in the effects of individual interventions, differences before and after each intervention were assessed by the paired *t* test or Wilcoxon test. LV and radial and femoral arterial dP/dt_max_ were compared using Bland-Altman analysis, corrected for multiple measurements per subject. Concordance between Ees and dP/dt_max_, defined as the percentage of data with agreement on the direction of change, was assessed by four-quadrant plots. Excellent concordance was assumed when the concordance rate was ≥ 90%. Linear mixed-effects model analysis was used to determine the contribution of the main cardiac variables (covariates: Ees, Ea, LV EDV and heart rate) to LV, femoral and radial dP/dt_max_. We also analyzed the impact of arterial factors (total vascular resistance (TVR) and arterial compliance (C_art_)) and preload-dependency (SVV) on dP/dt_max_ variables. Models were constructed using individual animals as subjects for random factors, and sequential experimental stages as repeated measurements. This allowed us to consider the correlation between subjects and non-constant variability over time, which is not considered by the standard linear regression analysis. A Toeplitz covariance structure was selected based on the corrected Akaike’s information criterion (AICc) value [18, 19], in which lower scores indicate superior fit [18, 19]. Model parameters were estimated using the restricted maximum likelihood method and the estimated fixed effect of each parameter was quantified by the estimated value (95% confidence interval).

A *P* value < 0.05 was considered statistically significant. All statistical analyses were two-tailed and performed using MedCalc Statistical Software version 17.4 (MedCalc Software bvba, Ostend, Belgium; https://www.medcalc.org; 2016) and SPPS (SPSS 21, SPPS Inc., Chicago, IL, USA).

## Results

### Hemodynamic changes during the experimental protocol

Hemodynamic variables throughout different experimental stages are detailed in Table 1 (afterload), Table 2 (preload), and Table 3 (contractility). As expected, MAP increased with phenylephrine by 38 ± 6% (Ea by 59 ± 23%) and decreased with nitroprusside by 32 ± 6% (Ea by 41 ± 11%). Preload modifications decreased EDV by 10 ± 9% with bleeding and increased it by 23 ± 16% after fluid bolus. During contractility changes, esmolol decreased LV dP/dt_max_ by 53 ± 11% (Ees by 36 ± 12%) and increased with dobutamine by 83 ± 17% (Ees by 54 ± 25%).

**Table 1:** Hemodynamic variables during afterload changes

	Phenylephrine		Sodium nitroprusside	
Variables	Before	After	Before	After
CO, L⋅min^− 1^	7.73 ± 1.40	7.07 ± 1.04^*^	8.09 ± 1.61	9.89 ± 1.49^*^
SV, ml	105 ± 11	96 ± 12^‡^	103 ± 11	119 ± 15^†^
HR, beats⋅min^− 1^	73 ± 11	73 ± 8	79 ± 11	75 ± 11^*^
MAP, mmHg	80 ± 9	111 ± 13^‡^	81 ± 8	55 ± 4^‡^
EDV, ml	218 ± 47	214 ± 45	211 ± 43	212 ± 45
ESV ml	110 ± 42	115 ± 41	105 ± 40	89 ± 43^†^
LV Ped, mmHg	13 ± 4	18 ± 4^‡^	12 ± 3	7 ± 3^‡^
LV Pes, mmHg	84 ± 11	122 ± 15^‡^	90 ± 12	60 ± 7^‡^
Ea, mmHg⋅ml^− 1^	0.69 ± 0.13	1.09 ± 0.20^‡^	0.77 ± 0.16	0.45 ± 0.09^‡^
Ees, mmHg⋅ml^− 1^	0.36 ± 0.12	0.48 ± 0.11^‡^	0.41 ± 0.14	0.35 ± 0.14 ^‡^
LV ejection fraction, %	52 ± 10	48 ± 9^*^	52 ± 10	60 ± 13^†^
SVV, %	10.9 ± 3.6	5.8 ± 2.3^‡^	10.9 ± 2.5	21.1 ± 5.9^†^
LV dP/dt_max_, mmHg⋅s^− 1^	1003 ± 158	1245 ± 175^‡^	1089 ± 157	963 ± 182^*^
Femoral dP/d_max_, mmHg⋅s^− 1^	306 ± 52	378 ± 53^†^	335 ± 75	226 ± 52^†^
Radial dP/dt_max_, mmHg⋅s^− 1^	480 ± 110	409 ± 69^*^	463 ± 125	321 ± 96^‡^

Data are presented as mean ± SD

*LV* left ventricle, *CO* cardiac output, *SV* stroke volume, *HR* heart rate, *MAP* mean arterial pressure, *EDV* left ventricular end-diastolic volume, *ESV* left ventricular end-systolic volume, *LV Ped* left ventricular end-diastolic pressure, *LV Pes* left ventricular end-systolic pressure, *Ees* end-systolic elastance, *Ea* effective arterial elastance, *SVV* stroke volume variation, *dP/dtmax* peak rate of pressure

*P < 0.05, ^†^*P* ≤ 0.001, ^‡^*P* ≤ 0.0001 vs “before” stage

**Table 2:** Hemodynamic variables during preload changes

	Bleeding		Fluid administration	
Variables	Before	After	Before	After
CO, L⋅min^−1^	7.87 ± 1.44	7.64 ± 1.32	7.89 ± 1.70	9.11 ± 2.42^*^
SV, ml	107 ± 12	108 ± 14	109 ± 15	118 ± 22^*^
HR, beats⋅min^− 1^	73 ± 10	71 ± 10	72 ± 10	76 ± 8^*^
MAP, mmHg	79 ± 11	56 ± 7^‡^	63 ± 9	78 ± 9^*^
EDV, ml	234 ± 50	211 ± 57^†^	215 ± 52	259 ± 47^†^
ESV ml	124 ± 48	100 ± 49^†^	104 ± 48	141 ± 45^†^
LV Ped, mmHg	12 ± 3	5 ± 4^‡^	7 ± 3	16 ± 4^†^
LV Pes, mmHg	83 ± 13	62 ± 8^‡^	71 ± 8	82 ± 10^*^
Ea, mmHg⋅ml^−1^	0.67 ± 0.14	0.54 ± 0.09^†^	0.59 ± 0.12	0.58 ± 0.15
Ees, mmHg⋅ml^− 1^	0.36 ± 0.13	0.39 ± 0.16	0.39 ± 0.15	0.31 ± 0.11^*^
LV ejection fraction, %	49 ± 11	55 ± 13^†^	54 ± 13	47 ± 10^*^
SVV, %	12.4 ± 3.3	25.7 ± 8.6^†^	19.8 ± 5.9	8.3 ± 3.9^*^
LV dP/dt_max_, mmHg⋅s^− 1^	993 ± 184	845 ± 218^†^	927 ± 195	1007 ± 49
Femoral dP/d_max_, mmHg⋅s^− 1^	297 ± 53	238 ± 63^†^	267 ± 63	279 ± 48
Radial dP/dt_max_, mmHg⋅s^− 1^	422 ± 178	313 ± 164^†^	376 ± 135	405 ± 111

Data are presented as mean ± SD

*LV* left ventricle, *CO* cardiac output, *SV* stroke volume, *HR* heart rate, *MAP* mean arterial pressure, *EDV* left ventricular end-diastolic volume, *ESV* left ventricular end-systolic volume, *LV Ped* left ventricular end-diastolic pressure, *LV Pes* left ventricular end-systolic pressure, *Ees* end-systolic elastance, *Ea* effective arterial elastance, *SVV* stroke volume variation*, dP/dtmax* peak rate of pressure

*P < 0.05, ^†^*P* ≤ 0.001, ^‡^*P* ≤ 0.0001 vs “before” stage

**Table 3:** Hemodynamic variables during contractility changes

	Esmolol		Dobutamine	
Variables	Before	After	Before	After
CO, L⋅min^−1^	8.87 ± 1.88	5.15 ± 1.09^‡^	8.16 ± 2.01	11.23 ± 3.25^‡^
SV, ml	116 ± 13	75 ± 11^‡^	111 ± 17	131 ± 22^‡^
HR, beats⋅min^− 1^	76 ± 10	69 ± 9^†^	73 ± 10	85 ± 15^†^
MAP, mmHg	73 ± 13	50 ± 4^‡^	71 ± 8	83 ± 10^‡^
EDV, ml	209 ± 33	200 ± 27	210 ± 28	208 ± 29
ESV ml	93 ± 28	125 ± 22^†^	97 ± 23	74 ± 28^‡^
LV Ped, mmHg	15 ± 4	13 ± 3^*^	14 ± 4	15 ± 4
LV Pes, mmHg	76 ± 15	53 ± 8^†^	73 ± 11	85 ± 12^‡^
Ea, mmHg⋅ml^−1^	0.53 ± 0.13	0.55 ± 0.12	0.54 ± 0.13	0.55 ± 0.15
Ees, mmHg⋅ml^− 1^	0.37 ± 0.10	0.23 ± 0.06^‡^	0.33 ± 0.07	0.50 ± 0.11^‡^
LV ejection fraction, %	56 ± 7	38 ± 5^‡^	38 ± 5	53 ± 7^‡^
SVV, %	9.4 ± 4.4	17.1 ± 3.6	10.1 ± 3.8	8.9 ± 4.2^*^
LV dP/dt_max_, mmHg⋅s^− 1^	1067 ± 220	485 ± 58^‡^	927 ± 174	1701 ± 384^‡^
Femoral dP/d_max_, mmHg⋅s^− 1^	292 ± 57	171 ± 44^‡^	287 ± 56	392 ± 70^‡^
Radial dP/dt_max_, mmHg⋅s^− 1^	401 ± 142	204 ± 66^‡^	391 ± 151	565 ± 192^‡^

Data are presented as mean ± SD

*LV* left ventricle, *CO* cardiac output, *SV* stroke volume, *HR* heart rate, *MAP* mean arterial pressure, *EDV* left ventricular end-diastolic volume, *ESV* left ventricular end-systolic volume, *LV Ped* left ventricular end-diastolic pressure, *LV Pes* left ventricular end-systolic pressure, *Ees* end-systolic elastance, *Ea* effective arterial elastance, *SVV* stroke volume variation, *dP/dtmax* peak rate of pressure

*P < 0.05, ^†^*P* ≤ 0.001, ^‡^*P* ≤ 0.0001 vs “before” stage

### Ees, LV and arterial dP/dt_max_ evolution

**Fig. 2:**
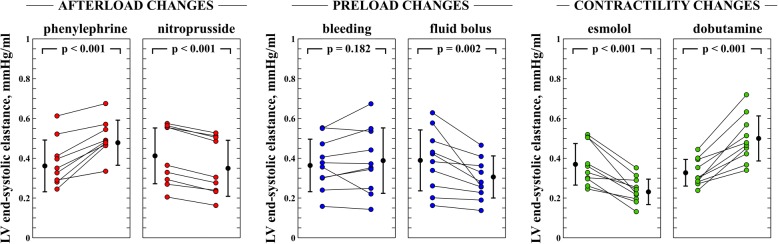
End-systolic elastance (Ees) evolution during different experimental conditions. Individual values of Ees during each experimental stage. Black points with bars represent the mean value and the standard deviation of changes in each experimental condition. Colored points represent individual changes in each animal. LV, left ventricle

**Fig. 3:**
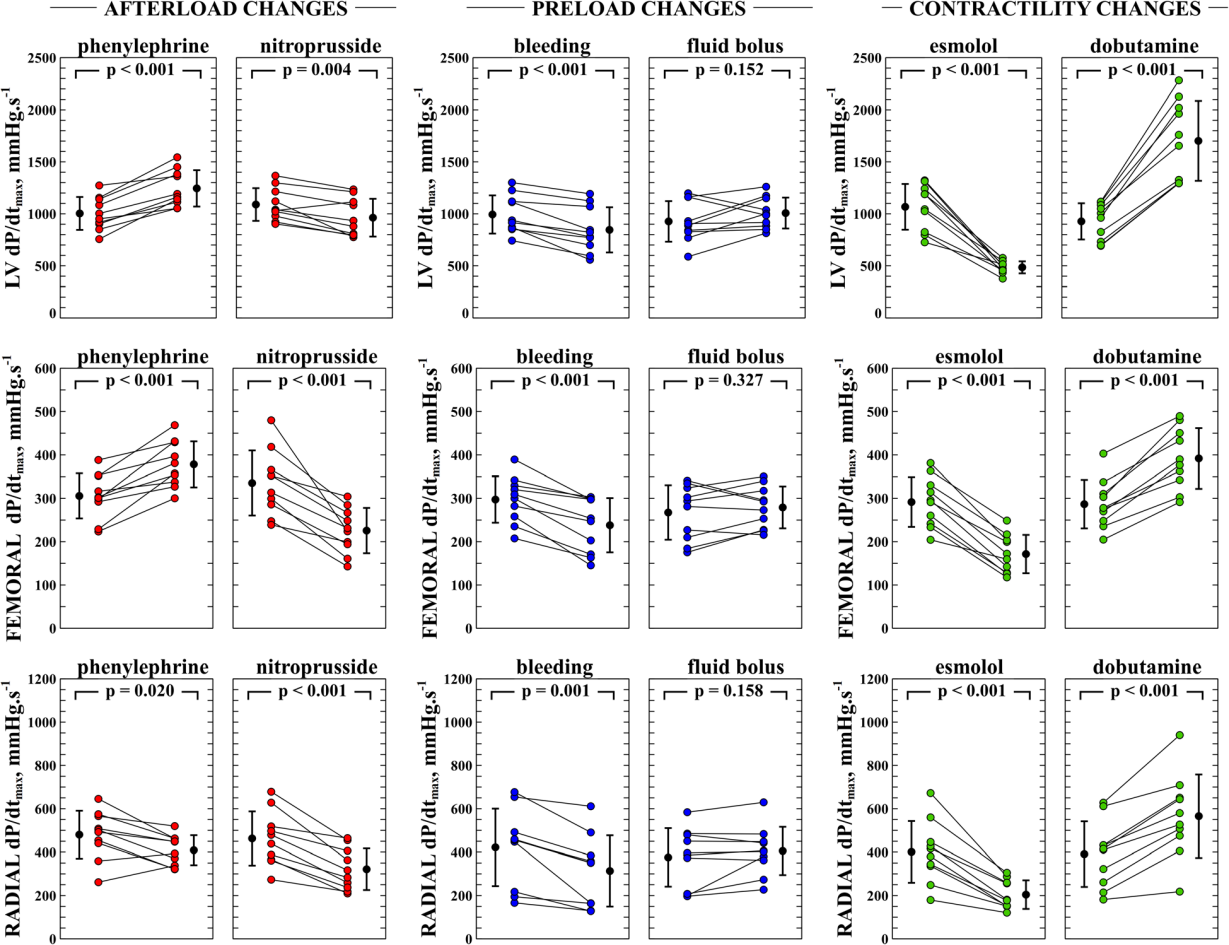
Evolution of left ventricular (LV) and arterial (femoral and radial) maximal rate of rise in pressure (dP/dt_max_) during different experimental conditions. Individual changes in different dP/dt_max_ variables during each experimental stage. Black circles with bars represent the mean value and the standard deviation of changes in each experimental condition. Colored circles represent individual changes in each animal

**Fig. 4:**
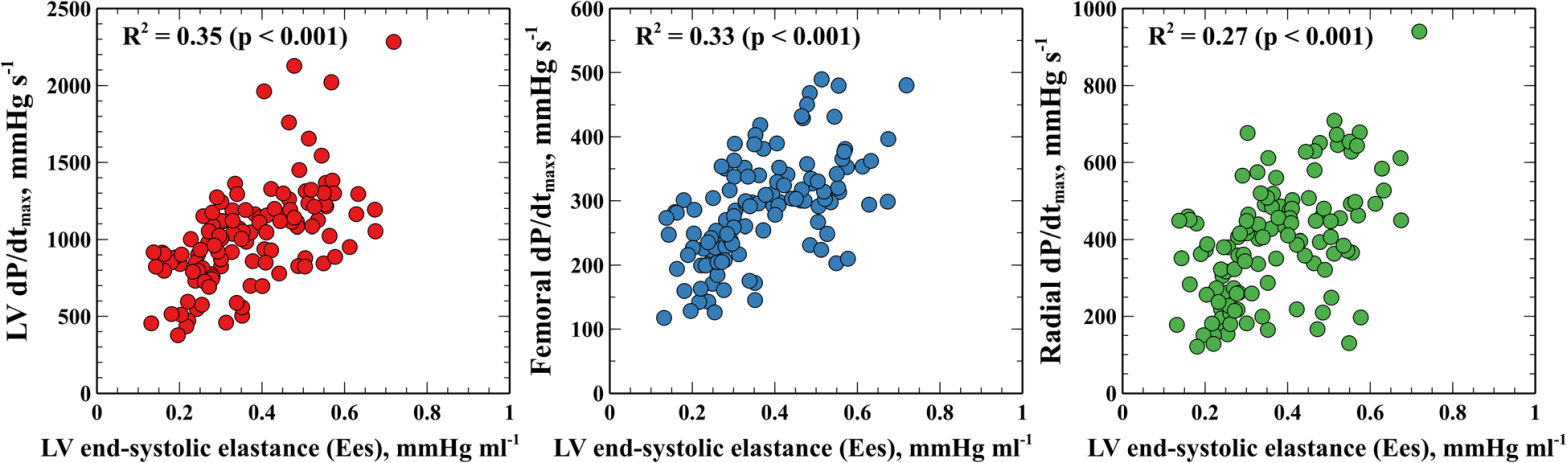
Relationship between left ventricular (LV) end-systolic elastance (Ees) and central and peripheral maximal rate of rise in pressure (dP/dt_max_). Naïve relationship (not considering between-subject and within-subject sources of variability) between LV Ees and LV, femoral and radial dP/dt_max_

**Fig. 5:**
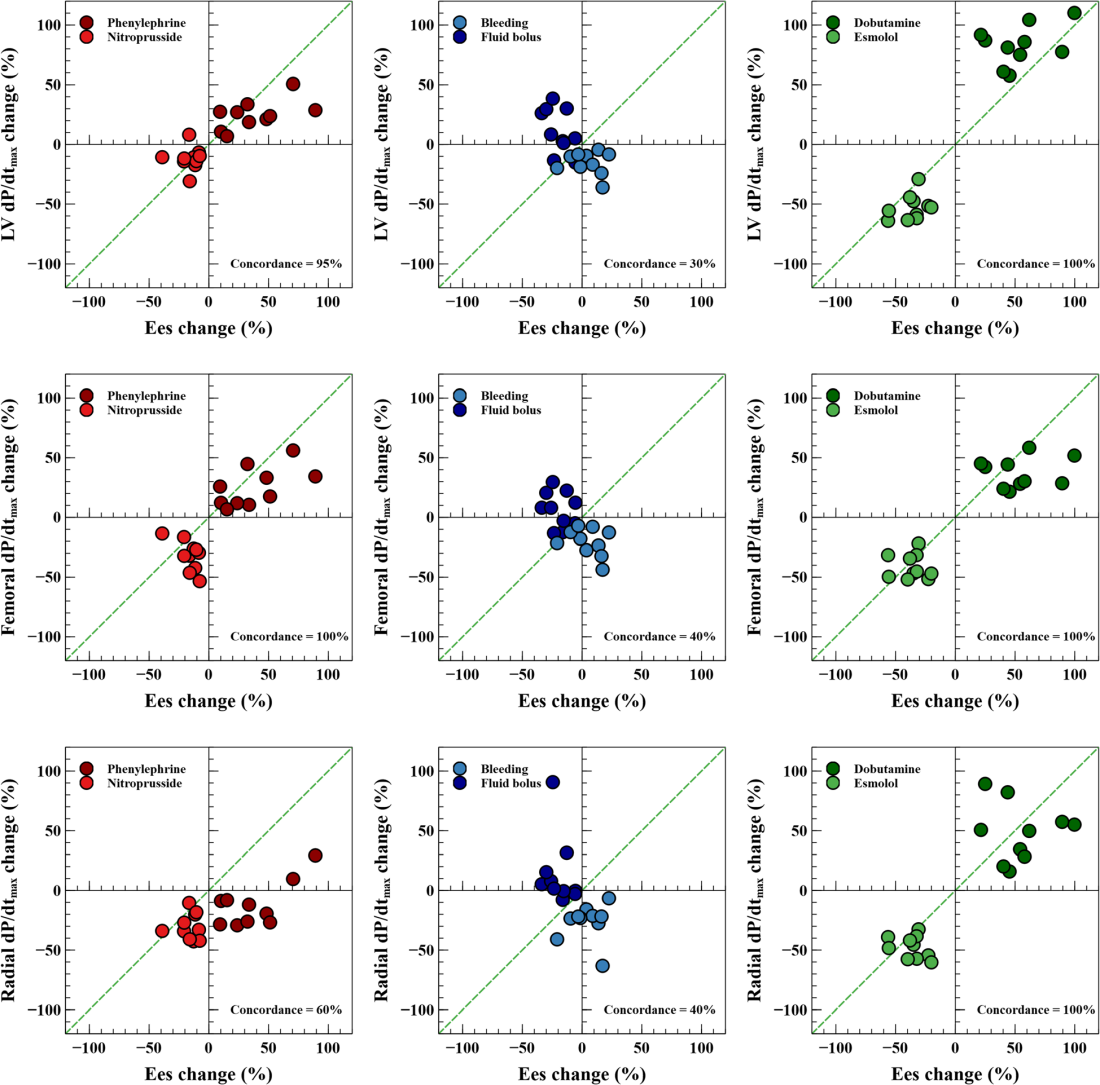
Concordance on percentage changes in left ventricular (LV), femoral and radial maximal rate of rise in pressure (dP/dt_max_) and percentage changes in end-systolic elastance (Ees) during the different experimental stages. Four-quadrant plots (concordance graphs) showing the relationship between percentage changes in LV Ees and LV and arterial dP/dt_max_ during each experimental condition. Good trending capability was assumed when most of the data lie in the right-upper and the left-lower quadrants. Dashed green line represents the line of equality

The individual changes in Ees, LV and arterial dP/dt_max_ during each experimental stage are shown in Figs. 2 and 3. Ees was significantly changed in all hemodynamic conditions, except during bleeding. The relationship between Ees and LV, femoral and radial dP/dt_max_ was *R*^2^ = 0.35, 0.33 and 0.27 (*P* <0.001) respectively (Fig. 4). Although the relationship between changes in LV and arterial dP/dt_max_ was good (*R*^2^ = 0.56 and 0.45 for femoral and radial dP/dt_max_; *P* < 0.0001, respectively, Additional file 1: Figure S1), arterial dP/dt_max_ was lower than LV dP/dt_max_ in all cases (radial dP/dt_max_ values were greater than femoral dP/dt_max_ values) (Additional file 1: Figure S2). Percentage changes in dP/dt_max_ and Ees had good concordance, especially LV and femoral dP/dt_max_ during afterload and contractility variations (Fig. 5). Adjusting LV and arterial dP/dt_max_ to EDV barely improved this trend in capability (Additional file 1: Figure S3).

### Influence of cardiac factors on LV and arterial dP/dt_max_

In the linear mixed model analysis, the main determinant of dP/dt_max_ variables was contractility (Ees) (Table 4). If holding other estimates constant, for an increase of 0.1 mmHg⋅ml^− 1^ in Ees, an absolute increase in LV dP/dt_max_ of 167.5 mmHg⋅s^− 1^, in femoral dP/dt_max_ of 32.6 mmHg⋅s^− 1^ and in radial dP/dt_max_ of 38.3 mmHg⋅s^− 1^ can be expected. Other factors, such as heart rate or EDV, also influenced dP/dt_max_, but to a much lesser degree. Cardiac afterload, when assessed using Ea, only had a statistically significant effect on femoral dP/dt_max_.

**Table 4:** Estimated values of fixed effects on left ventricular and peripheral dP/dt_max_ according to a linear mixed model analysis

	LV dP/dt_max_	Femoral dP/dt_max_	Radial dP/dt_max_
Cardiac factors			
Ees, mmHg ml^−1^	1674.7 (1394.9–1954.3)^‡^	326.2 (236.8–415.5)^‡^	382.9 (205.6–560.3)^‡^
Ea, mmHg ml^− 1^	5.6 (− 116.3–127.5)	59.9 (28.2–91.6)^†^	− 47.5 (− 113.2–18.1)
LV EDV, mL	1.2 (0.4–2.0)^*^	0.4 (0.2–0.7)^†^	0.7 (0.2–1.2)^†^
Heart rate, bpm	21.3 (17.7–24.9)^‡^	3.3 (2.1–4.5)^‡^	8.2 (6.1–10.3)^‡^
Arterial factors			
TVR, dyn cm s^− 5^		− 0.1 (− 0.2 – − 0.1)^‡^	−0.3 (− 0.4 – − 0.2)^‡^
C_art_, ml mmHg^− 1^		− 69.5 (− 89.7 – − 49.3)^‡^	−90.9 (− 112.2 – − 69.6)^‡^
Preload-dependency			
SVV, %	−7.46 (− 12.52 – − 2.40)^†^	−6.62 (− 8.07 – − 5.18)^‡^	−8.42 (− 9.83 – − 7.01)^‡^

Estimates are presented as estimated values (95% confidence interval). Estimates reflect the average change in the dependent variable per unit increase of the fixed effect

*LV* left ventricle, *Ees* left ventricular end-systolic elastance, *Ea* effective arterial elastance, *EDV* end-diastolic volume, *TVR* total vascular resistance, *C*_*art*_ arterial compliance, *SVV* femoral pulse pressure-derived stroke volume variation, *dP/dtmax* peak rate of pressure

*P < 0.05; ^†^*P* < 0.01; ^‡^*P* < 0.001

### Arterial factors influencing femoral and radial dP/dt_max_

We also analyzed the impact of arterial system properties (Ea, TVR and C_art_) on arterial dP/dt_max_. Both TVR and C_art_ affected peripheral dP/dt_max_: when TVR and C_art_ decreased, arterial dP/dt_max_ increased. However, the impact of C_art_ was markedly greater than TVR. Therefore, although changes in arterial load, as quantified by Ea and TVR, will produce opposite effects on dP/dt_max_, the overall result will eventually depend on the balance between the relative magnitude of these two factors. For example, a 10% increase in C_art_ will decrease radial dP/dt_max_ by 9 mmHg⋅s^− 1^, whereas a similar relative increase in TVR will reduce radial dP/dt_max_ by only 0.03 mmHg⋅s^− 1^ (Table 4).

### Influence of preload dependency on LV and arterial dP/dt_max_

When assessing the preload dependency, the impact of this factor was seen on all dP/dt_max_ indexes. The higher the preload dependency, the lower the arterial and LV dP/dt_max_. For example, an increase in SVV from 5% to 15% (if keeping other determinants constant) was associated with a decrease in LV dP/dt_max_ of 74.6 mmHg⋅s^− 1^, in femoral dP/dt_max_ of 66 mmHg⋅s^− 1^ and in radial dP/dt_max_ of 84 mmHg⋅s^− 1^.

## Discussion

Our study demonstrated that LV contractility, referenced to LV Ees, can be reasonably well-estimated and continuously tracked using the analysis of the peripheral arterial pressure waveform. Although other factors influenced femoral and radial dP/dt_max_, the main determinant of changes in peripheral dP/dt_max_ was LV Ees.

Adequate assessment of the LV inotropic state is an important component of the hemodynamic evaluation of critically ill patients [1]. LV systolic impairment is not only associated with primary cardiac affections, such as cardiogenic shock or chronic heart failure, but it has been increasingly acknowledged as a key phenomenon in the hemodynamic disorders described in septic shock and in perioperative cardiac complications in patients undergoing major cardiac and noncardiac surgery [2, 20, 21].

Ideally, the optimal index for evaluating LV contractility should be sensitive to changes in inotropism and insensitive to loading conditions. However, assessing LV contractility while theoretically isolating the heart from loading conditions can be challenging, especially in critically ill patients, where modifications of these factors are particularly evident due to the intrinsic evolution of the pathological process and the frequent use of vasoactive therapy that can affect both preload and afterload conditions [6, 22]. Although LV Ees is considered a load-independent marker for defining myocardial contractility [23, 24], its measure is mostly relegated to research studies because of the inherent invasiveness and technical difficulties in its measure. In clinical practice, LV systolic function is usually evaluated by calculating EF_LV_ using standard echocardiography. However, EF_LV_ has limitations as an index of intrinsic contractility, mostly related to its strong dependency on loading conditions and the assumptions about LV geometry [5, 25], which makes its interpretation difficult in the critically ill [6]. Unfortunately, even newer echocardiographic or thermodilution-derived indexes used to assess LV systolic function carry the same limitations or can be only used intermittently [26–29].

Left ventricular dP/dt_max_ has been traditionally used as a reliable marker of myocardial performance [8, 30]. Under normal conditions, the maximal rate of LV pressure is usually developed during isovolumetric contraction [30], so theoretically LV dP/dt_max_ should be relatively insensitive to afterload. In our study, LV dP/dt_tmax_ was not related to afterload changes according to the lack of influence of Ea. Previous studies have demonstrated that LV dP/dt_max_ remains independent of afterload within physiological levels of blood pressure, but it could be affected by large reductions in afterload [30–33]. On the other hand, because the arterial dP/dt_max_ occurs during the ejection phase just after the opening of the aortic valve, it is then exposed to the influence of the arterial system [10]. Furthermore, as the arterial pressure is recorded further away from the LV, the impact of arterial factors such as reflected pressure waves is more evident. Moreover, as dP/dt_max_ reflects the need for the pressure to grow in a given time at a constant diastolic pressure, the higher the systolic pressure to achieve, the potentially higher the dP/dt_max_. Therefore, the differences in the absolute magnitude observed between LV dP/dt_max_ and peripheral dP/dt_max_ should be interpreted under this perspective: the outflow of blood flow by definition reduces the absolute value of dP/dt_max_ measured at any arterial site, and the central-to-peripheral dP/dt_max_ gradient represents the tapering effect of the arterial system, buffering the intraventricular pressure into a more compliant system [9, 10], while a greater radial dP/dt_max_ may represent the impact of a lower compliance and the earlier wave reflections on the systolic part of the arterial pressure waveform [34]. However, despite the effect of the arterial system properties on the dP/dt_max_, its contribution was significantly lower when compared with the prominent impact of contractility changes. Moreover, our data suggest that femoral dP/dt_max_ may be used even in patients with vasoplegia or treated with vasoactive agents.

The sensitivity of LV dp/dt_max_ to changes in preload has been extensively described [30–32, 35]. In our study, the impact of EDV was small but especially relevant during isolated modifications in preload, which it is in agreement with previous experimental and clinical studies [30–32, 35, 36]. However, when considering the effects of preload, adjusting LV and arterial dP/dt_max_ to EDV, the trending capability of both LV and arterial dP/dt_max_ did not improve significantly, which reinforces the limited influence of preload on dP/dt_max_ changes. We also confirmed that LV and arterial dP/dt_max_ were affected by preload-dependency status [9]. As SVV is a compound variable depending on the preload status and the cardiac function [37], its influence should be interpreted by the combined interaction of these factors.

The present study indicates that the main determinant of changes in both LV and arterial dP/dt_max_ throughout the study was LV contractility. Our results are consistent with previous studies comparing arterial dP/dt_max_ with other surrogates of LV contractility [9–11]. However, most of the previous studies compared the arterial dP/dt_max_ with LV dP/dt_max_ or used an in vitro experimental preparation. In our study, we compared LV and peripheral dP/dt_max_ in vivo with the gold-standard index of LV contractility while studying a wide range of hemodynamic conditions, compromising two-way changes in preload, afterload and contractility. Moreover, we also identified and quantified the potential influence of several cardiac and arterial factors, so the continuous estimation of the actual LV contractility could be further improved considering the effect of the most prominent factors.

### Clinical usefulness and limitations

Arterial catheterization and arterial pressure monitoring are part of the usual care in both critically ill patients with hemodynamic instability and patients undergoing high-risk surgery. Complementary information on LV contractility, as provided by real-time monitoring of arterial dP/dt_max_, may add valuable information on the dynamic cardiac function assessment of these patients over time and on treatments. Moreover, the combined data derived from the analysis of the arterial pressure, such as preload dependency and cardiac output, in combination with the continuous assessment of contractility by arterial dP/dt_max_, could add a comprehensive evaluation of the hemodynamic status and may help to improve future resuscitation algorithms.

A few limitations should be considered when interpreting our results. First, LV and arterial dP/dt_max_ should be interpreted with caution in patients with aortic valvular disease or the presence of LV tract obstruction. Aortic stenosis or dynamic LV tract obstruction, for example, creates a significant pressure gradient over the aortic valve, and thus a large difference between LV dP/dt_max_ and arterial dP/dt_max_. Second, peripheral-to-central decoupling in arterial pressure, as described during septic shock [38], may alter the relationship between LV and arterial dP/dt_max_, so our results may differ in septic shock conditions. Third, our experimental protocol involved many different hemodynamic conditions, and these modifications were sequentially generated or could be not fully represented, as during bleeding stage, where the hemodynamic changes were partially compensated. Fourth, although we have carefully checked the arterial pressure signal quality, we used fluid-filled catheters for assessing arterial dP/dt_max_ instead of pressure-tipped catheters, which are known to be exposed to overdamping/underdamping phenomena. Finally, our study was performed on healthy pigs submitted to anesthesia with known cardiovascular effects, so our results should be interpreted with caution when extrapolating to human cardiovascular physiology.

## Conclusion

Although arterial dP/dt_max_ is a complex function subject to central and peripheral factors, both radial and particularly femoral dP/dt_max_ allowed reasonably good tracking of LV contractility changes during different loading and inotropic conditions across all domains of vasomotor tone, contractility and volume status. Therefore, real-time assessment of LV contractility may be evaluated in clinical practice by monitoring peripheral arterial dP/dt_max_.

## Additional file

Additional file 1: **Figure S1.** Relationship between left ventricular and peripheral (femoral and radial) dP/dt_max_. **Figure S2.** Bland-Altman analysis (corrected for multiple measurements per subject) between left ventricular and peripheral (femoral and radial) dP/dt_max_. **Figure S3.** Concordance on percentage changes in left ventricular, femoral and radial dP/dtmax and percentage changes in end-systolic elastance (Ees) during the different experimental stages. (DOCX 726 kb)

## Abbreviations

AICc: Akaike’s information criterion
C_art_: Arterial compliance
CO: Cardiac output
dP/dt_max_: Maximal rate of rise in pressure
E: Elastance
Ea: Effective arterial elastance
EDV: Left ventricular end-diastolic volume
Ees: End-systolic elastance
EF_LV_: Left ventricular ejection fraction
ESV: Left ventricular end-systolic volume
ICU: Intensive care unit
IVC: Inferior vena cava
LV: Left ventricle
MAP: Mean arterial pressure
Pes: Left ventricular end-systolic pressure
PV: Pressure-volume
SV: Stroke volume
SVV: Stroke volume variation
TVR: Total vascular resistance
V_0_: Left ventricular unstressed volume
