# Supplementary material for: Performance comparison of ventricular and arterial dP/dtmax for assessing left ventricular systolic function during different experimental loading and contractile conditions
Source: Crit Care. 2018 Nov 29;22:325. doi: 10.1186/s13054-018-2260-1 (PMC6262953; doi:10.1186/s13054-018-2260-1)
Supplement: Supplementary file 1 — Figure S1. Relationship between left ventricular and peripheral (femoral and radial) dP/dtmax. Figure S2. Bland-Altman analysis (corrected for multiple measurements per subject) between left ventricular and peripheral (femoral and radial) dP/dtmax. Figure S3. Concordance on percentage changes in left ventricular, femoral and radial dP/dtmax and percentage changes in end-systolic elastance (Ees) during the different experimental stages. (DOCX 726 kb) [file 13054_2018_2260_MOESM1_ESM.docx]

**Performance comparison of ventricular and arterial dP/dt_max_ for assessing left ventricular systolic function during different experimental loading and contractile conditions**

Manuel Ignacio MONGE GARCIA^1^, Zhongping JIAN^2^, Jos J. SETTELS^2^, Charles HUNLEY^3^, Maurizio CECCONI^4^, Feras HATIB^2^, Michael R. PINSKY^5^

1. Unidad de Cuidados Intensivos, Hospital SAS de Jerez, Jerez de la Frontera, Spain
2. Edwards Lifesciences, Irvine, California, USA.
3. Orlando Regional Medical Center, Orlando Health, Florida, USA.
4. Department Anaesthesia and Intensive Care Units, Humanitas Research Hospital, Humanitas University, Milan, Italy.
5. Department of Critical Care Medicine, University of Pittsburgh School of Medicine, Pittsburgh, USA.

**Additional File**

**Supplementary Material**

| **Figure S1. Relationship between left ventricular and peripheral (femoral and radial) dP/dt_max_.** |
| --- |
| 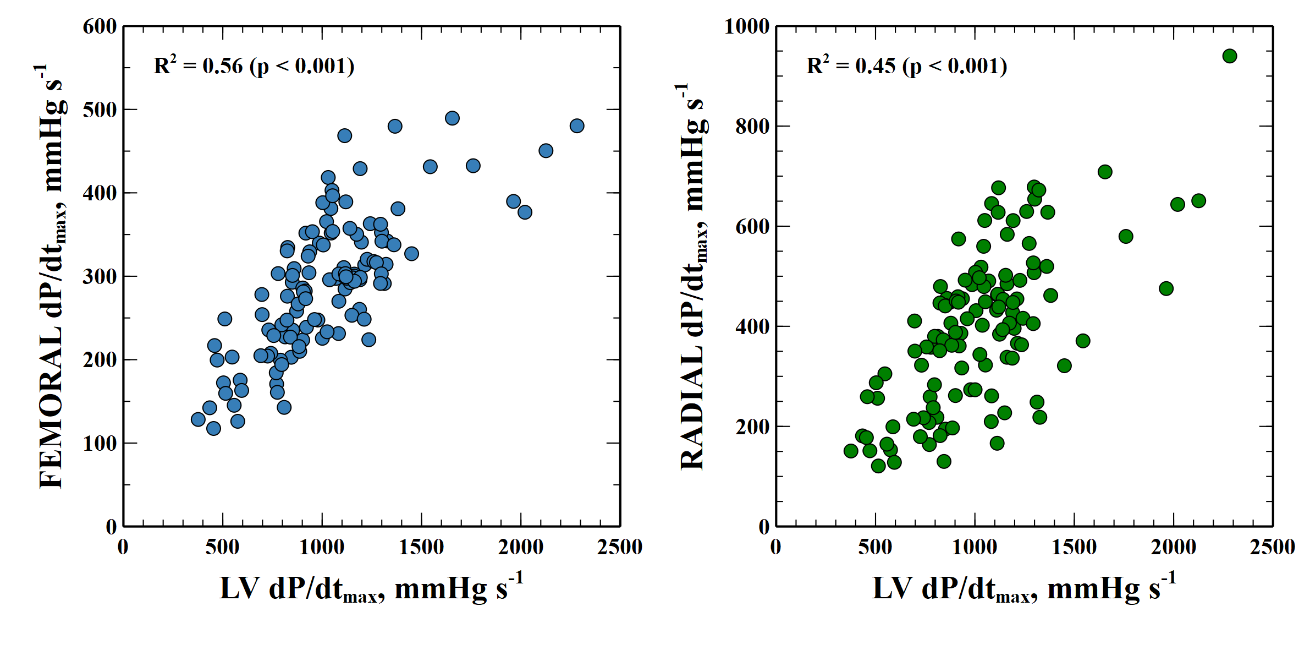 |
| LV: left ventricular. |

| **Figure S2. Bland-Altman analysis (corrected for multiple measurements per subject) between left ventricular and peripheral (femoral and radial) dP/dt_max_.** |
| --- |
|    |
| LV: left ventricular. |

| **Figure S3. Concordance analysis for changes in left ventricular, femoral and radial dP/dt_max_ and percentage changes in end-systolic elastance (Ees) during the different experimental stages.** |
| --- |
| 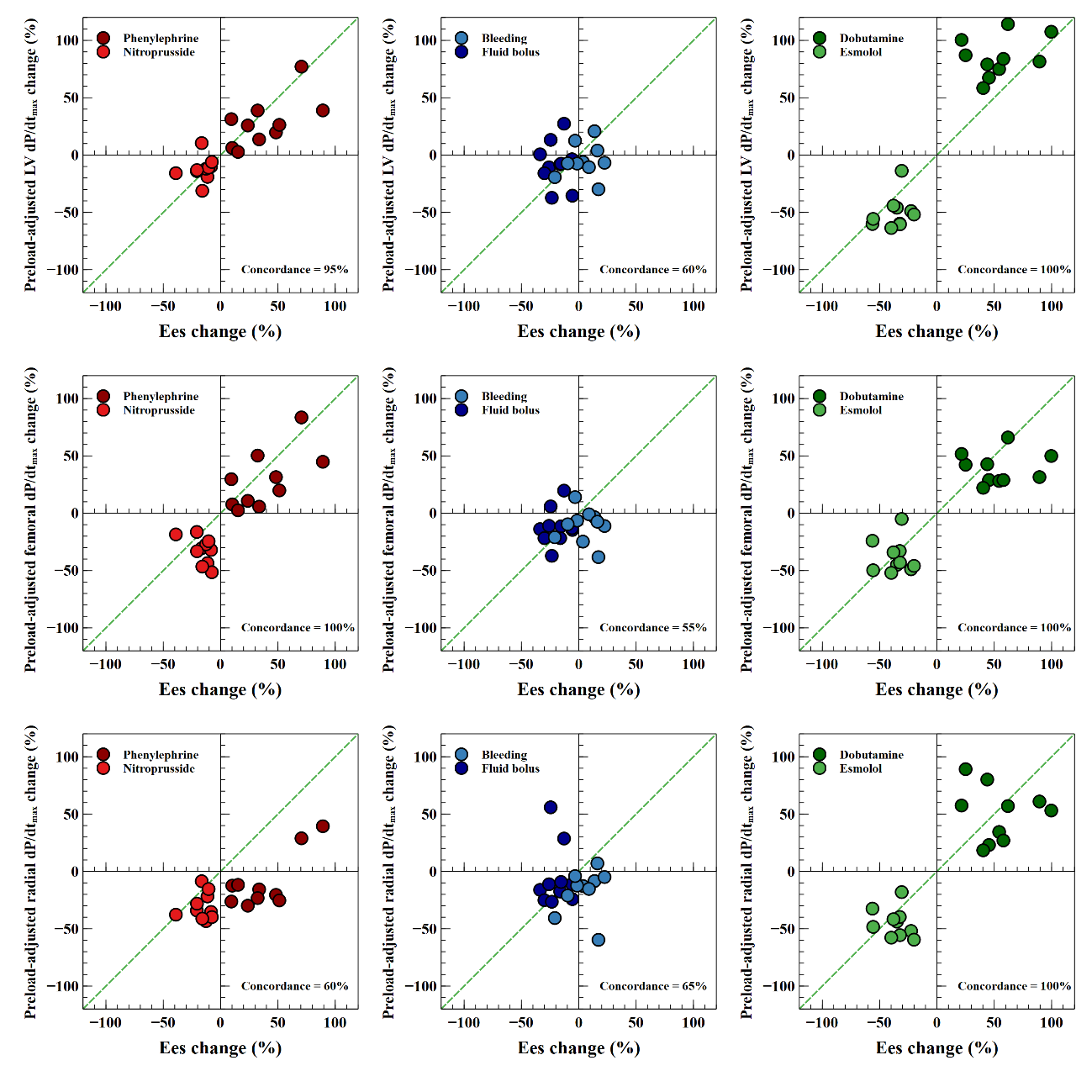 |
| Four-quadrant plots (concordance graphs) showing the relationship between percentage changes in LV end-systolic elastance (Ees) and preload-adjusted LV and arterial dP/dt_max_ during each experimental condition. Good trending capability was assumed when most of the data lie in the right-upper and the left-lower quadrants. Adjusting preload was performed dividing dP/dt_max_ by left ventricular end-diastolic volume. Dashed green line represents the line of equality. |
